# What is the Golgi apparatus, and why are we asking?

**DOI:** 10.1186/1741-7007-9-63

**Published:** 2011-09-30

**Authors:** Sean Munro

**Affiliations:** 1 MRC Laboratory of Molecular Biology, Hills Road, Cambridge CB2 0QH, UK

## Don't we all know what the Golgi apparatus is?

**Figure 1:**
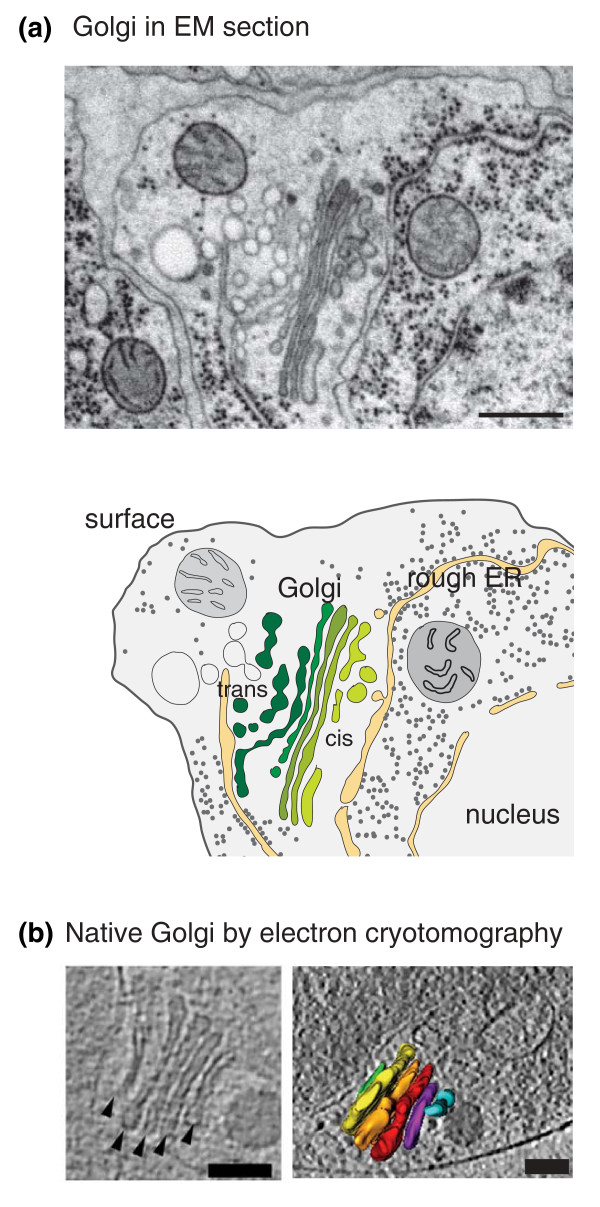
**What the classical Golgi looks like**. **(a) **Top: an electron micrograph of a section through a typical metazoan cell. The Golgi is a stack of cisternae arranged from cis (light green) to trans (dark green). Note the contact sites between the trans cisternae and ER (*Caenorhabditis elegans *hypodermal cells courtesy of Gillian Howard (MRC-LMB); scale bar = 500 nm). Bottom: a schematic drawing of the electron micrograph on the left, with the important structures labeled: the cis-Golgi is colored pale green, the trans-Golgi dark green. **(b) **A Golgi from the unicellular green alga *Ostreococcus tauri *imaged by electron cryotomography. The sample is frozen rather than fixed and so this represents one of the first images of a native Golgi stack. Left: a single slice through the three-dimensional reconstruction. Right: the Golgi highlighted in a view through the whole cell: cisternae are colored purple, red, gold, yellow, and green (cis to trans), and ER light blue. From Henderson *et al. *(2007), *PLoS One *2, e749; scale bars = 100 nm.

Yes and no. The Golgi apparatus (or Golgi to its friends) is named after Camillo Golgi, who first reported in 1888 a reticular structure in the cytoplasm of many cell types that he found by silver chromate staining. The text book story, which most people probably do know, emerged with the advent of electron microscopy (EM) more than half a century later, when the structure was revealed to be a set of flattened membrane-bound compartments, or cisternae, that are typically arranged in a stack (Figure 1). Radiolabeling studies then led to the current dogma that the Golgi is the organelle through which newly made secretory and membrane proteins pass as they move from the endoplasmic reticulum (ER) to the plasma membrane, or other membrane-bound compartments of the cell; and it is now also part of the classic picture that the Golgi elaborates and edits the generic glycan structures that are attached to proteins in the ER.

## That all sounds very straightforward - what's the mystery?

**Figure 2:**
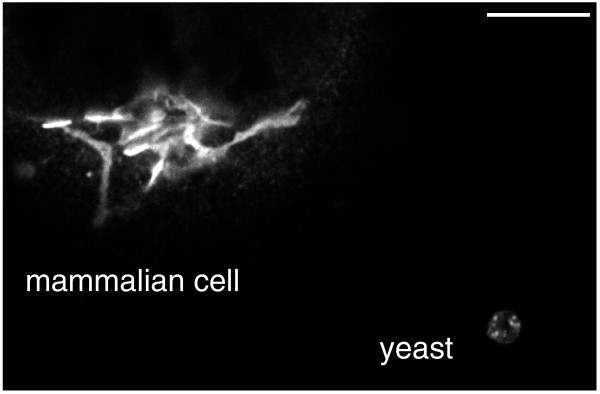
**How the Golgi differs between mammals and yeast**. Confocal immunofluorescence image of a mammalian cell and a yeast cell expressing the same myc-tagged Golgi protein (yeas tKre2). The mammalian Golgi stack forms ribbons next to the nucleus, whereas in yeast the Golgi comprises small cisternae found throughout the cytoplasm. Scale bar = 10 μm.

Well, first of all, the Golgi is by no means always as described by its discoverer. The structure that Camillo Golgi first observed was a typical mammalian Golgi, with individual stacks that are linked to form long connected ribbons arranged around the microtubule organizing center near the nucleus. However, in some cell types - for example muscle - and in most invertebrates and plants, the Golgi is not a linked ribbon but rather exists as one to hundreds of individual stacks scattered through the cytosol. Worse still, some species lack altogether the classic 'stack of plates' arrangement of the cisternae. This led to the belief that some species - fungi in particular - had no Golgi apparatus, and suggestions that the organelle emerged only after the first eukaryotes. However, it transpired that in these fungi the Golgi cisternae are present but spend most, and perhaps all, of their time apart. In more extreme cases such as microsporidia the Golgi is no more than a cluster of tubes and vesicles. Figure 2, which shows an immunofluorescence image of the Golgi in a mammalian and a yeast cell, illustrates the different arrangement of the membranes. The current view is that all eukaryotic cells have a Golgi of some sort, and thus it was a feature of the last common eukaryotic ancestor.

## Do these structural differences mean that the Golgi has distinct functions in different cells?

**Figure 3:**
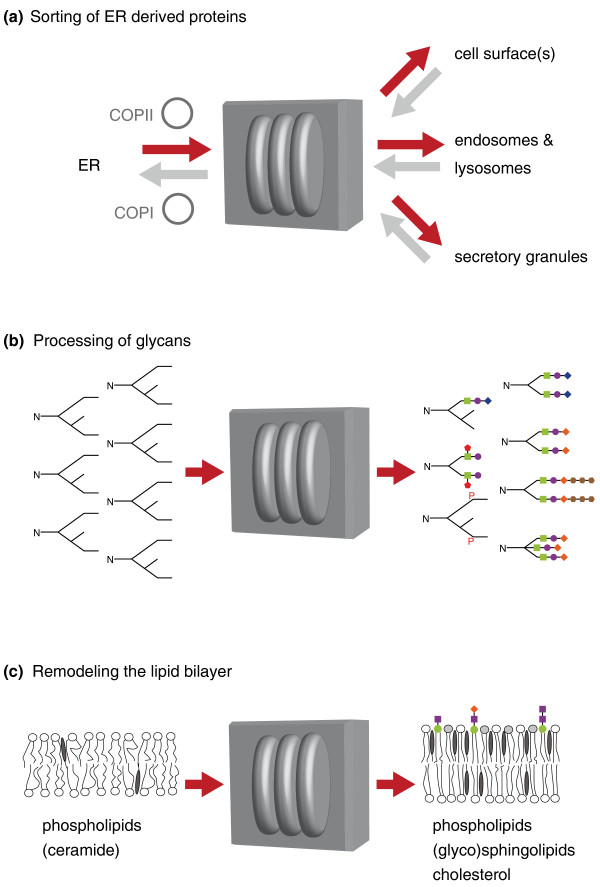
**What the Golgi does**. From the perspective of the cell, the Golgi can be thought of as a black box with material entering from ER or endosomes, and then leaving with various consequences. **(a) **Sorting. Newly made secreted and membrane proteins arrive at the cis-Golgi from the ER in COPII-coated vesicles and are sorted from the trans-Golgi to the other organelles of the cell. COPI vesicles retrieve escaped ER residents, and are widely, but not universally, thought to also recycle Golgi enzymes from later to earlier compartments as the cisternae mature. **(b) **Modification of proteins. Newly made proteins from the ER receive a range of post-translational modifications as they move through the Golgi stack, as illustrated here by the processing of N-linked glycans. **(c) **Modification of the lipid bilayer. The membrane of the ER is primarily composed of phospholipids. Sphingolipids, such as sphingomyelin in mammals, and glycolipids are made in the Golgi using ceramide delivered from the ER, and cholesterol levels also increase toward the trans side. Thus, the membrane leaving from the trans-Golgi is very different in composition to that which arrived from the ER.

Yes and no - again. Despite varying greatly in shape and size, the Golgi performs some roles that are almost certainly shared by all cells and species. All eukaryotes make their membrane and secreted proteins in the ER, and require some mechanism for routing them out of the ER to various destinations inside the cell, or out of it. One key role of the Golgi is as the main sorting point for all of this post-ER traffic (Figure 3a). Proteins for different destinations all exit the ER in vesicles coated in the specialized coat protein COPII, and these fuse to each other and to the first, or 'cis', cisterna of the Golgi. ER resident proteins that have escaped in the COP II vesicles are then recycled back to the ER in COPI vesicles, and the remaining proteins exit from the opposite, or 'trans', side of the Golgi. These proteins must be sorted into carriers to be transported to different destinations. Secreted and cell surface proteins are transported to the plasma membrane, whilst lysosomal proteins are initially sorted to endosomes that mature and subsequently fuse with lysosomes. All this departing traffic depends on specialized cargo receptors and traffic machinery that are recycled and returned to the Golgi, probably at the trans cisterna.

## So Golgi membranes don't all look the same, but they are all doing the same things?

Not exactly the same, no. All cells require that the Golgi provides the traffic route from the ER to the rest of the cell, but the Golgi also performs functions that are more variable between cell types. First, some cells make specialized secretory structures, such as the insulin-containing granules of the pancreas, and these too form at the Golgi (Figure 3a). Second, the Golgi adds post-translational modifications to the cargo proteins traveling through from the ER. The most prominent of these activities is the trimming and extension of the glycan core structures that are attached in the ER (Figure 3b). The precise glycan structures attached vary between protein, cell type and species, and they are synthesized by resident enzymes that are often arranged in the stack in the order in which they act. The diversity of glycosylation is illustrated by human blood groups, which arise from different alleles of a particular Golgi glycosyltransferase. In some cells long polymers of glycans are attached, and the Golgi has a major role in the biosynthesis of proteoglycans in animals and pectins in plants. Other modifications include sulfation, phosphorylation and proteolysis, and all of the enzymes involved are integral membrane proteins. This is strikingly distinct from the ER, where many of the resident proteins are soluble within the ER lumen, and may account for one morphological specialization of the Golgi membranes: it may be that the flattened shape of the cisternae reflects the need to keep the soluble cargo proteins in the lumen close to the 'catalytic carpet' of enzymes that lines Golgi membranes and applies the appropriate modifications to passing cargoes.

A further role for the Golgi is in lipid metabolism (Figure 3c). In particular, the Golgi contains enzymes that convert ceramide made in the ER into sphingolipids. In mammals these are sphingomyelin and glycosphingolipids, which are abundant components of the plasma membrane. These lipids have the capacity in model systems to combine with cholesterol to form domains, which has led to suggestions that they could contribute to protein sorting in the Golgi. It was noted years ago that the Golgi is able to direct sphingolipids to the plasma membrane rather than back to the ER, and it is tempting to speculate that this remodeling of the lipid bilayer may be a primordial function of the Golgi.

## Apart from evolutionary and morphological questions, this all sounds pretty well established - what are the issues?

Where shall I start? Perhaps the most recent issue - and one of the most controversial - is the so-called Golgi bypass. The classical view is that all protein traffic from the ER is directed through the Golgi apparatus. But a couple of papers have recently claimed that particular membrane proteins can bypass the Golgi and arrive at the cell surface with modifications acquired in the ER but lacking subsequent Golgi modification. The nature, and even existence, of this 'unconventional' secretion route is not universally agreed upon, and why only some proteins would be able to access such a route is unclear. The evidence for unconventional secretion will become more robust if and when its specific machinery is identified - especially given that the principal protein claimed so far to be required for bypassing the Golgi, GRASP, is itself a Golgi resident with a well established role in cisternal stacking.

## So what do we know about the specific machinery for conventional, dogma-respecting traffic through the Golgi?

Ah well. That is a rather embarrassing question. Although we know what arrives and departs, how proteins move from one side of the Golgi to the other has been debated for decades, and still is. The original model from electron micrographs was that cisternae formed on the cis side, and then 'progressed' or 'matured' through the stack until they broke up at the trans side or fused with a pre-existing trans cisterna. This was then challenged by the proposal that transport vesicles carry cargo forward through the stack. Although cisternal maturation is back in favor with most in the field, some recent papers have suggested that tubular connections form between cisternae to allow rapid forward movement of cargo. For a lucid account of the details of this long-running debate the interested reader is referred elsewhere (see below). It also remains to be seen if the nature of intra-Golgi transport has any implications for the way that the Golgi performs its fundamental roles for the cell.

A particularly poorly understood aspect of the Golgi is the generation of the carriers that move from the trans Golgi to the plasma membrane. Unlike other traffic steps, no coat proteins have been identified, and there may be redundant pathways to the plasma membrane, especially in polarized cell types such as epithelia and neurons where proteins need to be delivered to different parts of the cell surface. Likewise, there appear to be multiple routes back to the Golgi from endosomes, but how many routes, what machinery acts for which, and where they arrive at the Golgi are not yet resolved.

## Is that it for unresolved issues?

It's not. Another is the mechanism that ensures that the Golgi resident enzymes remain in the stack rather than departing with exiting cargo. There is evidence that transmembrane domains can contribute to retention, but how different enzymes are targeted to different parts is not known, or even how the transmembrane domains act.

How the Golgi stack is assembled from individual cisternae is also not well understood, nor the purpose that is served by the stacked arrangement, given that it is not a universal feature of all Golgis. Indeed, the issue of how and why the Golgi varies between organisms is also waiting to be resolved as more of the field explores species outside of the two kingdoms of lab life, yeast and HeLa cells.

Some lipid transport may also occur via non-vesicular routes, as contact sites can often be seen between the trans-Golgi and ER (see, for example, Figure 1a), and some sterol and ceramide transport proteins have binding domains for both organelles. However, the components and ubiquity of these contacts remain enigmatic. In mammalian cells, the Golgi ribbon and stacks fragment during mitosis to facilitate equal distribution between daughters, indicating that Golgi structure can be regulated. Finally there are the general issues of homeostasis and scaling that apply to all cellular structures (as discussed recently in *BMC Biology *by Wallace Marshall). In the case of the Golgi there must be homeostatic mechanisms underlying the stack's highly regular, if species-specific, size and shape.

## Is there hope for resolution of the issues in Golgi structure and function?

Max Planck argued that a scientific truth only triumphs when its opponents eventually die, but I believe that technological advances will save us from what would otherwise, I hope, be a long wait. In particular, recent advances in super-resolution microscopy hold the promise of clearly resolving the distribution of cargo, resident enzymes, and traffic machinery within individual cisternae, and even following this through time in living cells. In addition, improved methods of specimen preparation for electron microscopy, combined with tomography, provide new opportunities for understanding Golgi structure (Figure 1b). It can be hard to localize specific proteins in thick sections, but there has been exciting recent progress in studying frozen unfixed sections in which it may eventually be possible to recognize protein density corresponding to recently solved structures of trafficking components. Ultimately, a molecular level understanding of mechanism will need to move beyond descriptive studies towards the biochemical reconstitution of Golgi function *in vitro*. This is a daunting challenge that few, if any, labs are currently embracing, but it may be that particular steps can be addressed in isolation as structural biologists have recently had considerable success expressing recombinant membrane transport components that will be invaluable for such *in vitro *assays.

## Can't genetics help?

Much of the molecular machinery of the Golgi was identified by biochemical assays and yeast genetics. More recently, genome-wide RNA interference screens in metazoan cells have identified a few further components that were missed or were absent from yeast. More unexpectedly, an increasing number of rare genetic diseases are being found to be caused by null alleles of genes encoding Golgi proteins. It seems that the loss of some Golgi proteins that are ubiquitously expressed and well conserved in evolution results in defects that, although severe, are not cell lethal but instead specific for particular tissues or cargo proteins, or result in reduced levels of glycosylation. This suggests that some Golgi machinery is not required for basic traffic but for ensuring that the organelle functions at maximum efficiency, especially when large amounts of material are being secreted.

It is clear that much of the Golgi remains mysterious over 110 years after its discovery, a reflection surely of its complexity rather than the quality of research in the field. Given its central role in membrane traffic and the increasing number of links to human disease, resolving these questions seems certain to open the door to a new understanding of fundamental mechanisms in eukaryotic biology.

## Where can I find out more?

Emr S, Glick BS, Linstedt AD, Lippincott-Schwartz J, Luini A, Malhotra V, Marsh BJ, Nakano A, Pfeffer SR, Rabouille C, Rothman JE, Warren G, Wieland FT: **Journeys through the Golgi--taking stock in a new era**. *J Cell Biol *2009, **187:**449-453.

Giuliani F, Grieve A, Rabouille C: **Unconventional secretion: a stress on GRASP**. *Curr Opin Cell Biol *2011, **23:**498-504.

Glick BS, Luini A: **Models for Golgi traffic: a critical assessment**. *Cold Spring Harb Perspect Biol *2011. doi: 10.1101/cshperspect.a005215.

Hughson FM, Reinisch KM: **Structure and mechanism in membrane trafficking**. *Curr Opin Cell Biol *2010, **22:**454-460.

Lowe M: **Structural organization of the Golgi apparatus**. *Curr Opin Cell Biol *2011, **23:**85-93.

Marshall WF: **Origins of cellular geometry**. *BMC Biol *2011, **9:**57.

Smits P, Bolton AD, Funari V, Hong M, Boyden ED, Lu L, Manning DK, Dwyer ND, Moran JL, Prysak M, Merriman B, Nelson SF, Bonafé L, Superti-Furga A, Ikegawa S, Krakow D, Cohn DH, Kirchhausen T, Warman ML, Beier DR: **Lethal skeletal dysplasia in mice and humans lacking the golgin GMAP-210**. *N Engl J Med *2010, **362:**206-216.

Toomre D, Bewersdorf J: **A new wave of cellular imaging**. *Annu Rev Cell Dev Biol *2010, **26:**285-314.

van Meer G, Voelker DR, Feigenson GW: **Membrane lipids: where they are and how they behave**. *Nat Rev Mol Cell Biol *2008, **9:**112-124.

